# Smart Textile Technology for the Monitoring of Mental Health

**DOI:** 10.3390/s25041148

**Published:** 2025-02-13

**Authors:** Shonal Fernandes, Alberto Ramos, Mario Vega-Barbas, Carolina García-Vázquez, Fernando Seoane, Iván Pau

**Affiliations:** 1 Facultad de Diseño y Tecnología, University of Design, Innovation and Technology, 28016 Madrid, Spain; shonal.fernandes@udit.es (S.F.); alberto.ramos_serrano@hb.se (A.R.); carolina.garcia@udit.es (C.G.-V.); 2 ETSIS de Telecomunicación, Universidad Politécnica de Madrid, Calle Nikola Tesla S/N, 28038 Madrid, Spain; mario.vega@upm.es (M.V.-B.); ivan.pau@upm.es (I.P.); 3 Textile Materials Technology, Department of Textile Technology, Faculty of Textiles, Engineering and Business Swedish School of Textiles, University of Borås, 503 32 Boras, Sweden; 4 Institute for Clinical Science, Intervention and Technology, Karolinska Institutet, 141 83 Stockholm, Sweden; 5 Department of Medical Care Technology, Karolinska University Hospital, 141 57 Huddinge, Sweden; 6 Department of Clinical Physiology, Karolinska University Hospital, 141 57 Huddinge, Sweden

**Keywords:** mental health, smart textiles, monitoring, respiration rate, electroencephalogram, electrodermal activity, electrocardiogram, cortisol

## Abstract

In recent years, smart devices have proven their effectiveness in monitoring mental health issues and have played a crucial role in providing therapy. The ability to embed sensors in fabrics opens new horizons for mental healthcare, addressing the growing demand for innovative solutions in monitoring and therapy. The objective of this review is to understand mental health, its impact on the human body, and the latest advancements in the field of smart textiles (sensors, electrodes, and smart garments) for monitoring physiological signals such as respiration rate (RR), electroencephalogram (EEG), electrodermal activity (EDA), electrocardiogram (ECG), and cortisol, all of which are associated with mental health disorders. Databases such as Web of Science (WoS) and Scopus were used to identify studies that utilized smart textiles to monitor specific physiological parameters. Research indicates that smart textiles provide promising results compared to traditional methods, offering enhanced comfort for long-term monitoring.

## 1. Introduction

The World Health Organization (WHO) defines mental well-being as a state of mind that allows people to deal with the stresses of life and contribute to society in a way that brings value. Mental health takes various forms, and no single treatment works for everyone. The disorder exists on a spectrum, with the severity of disabilities varying based on factors such as the individual, age, gender, and environment. It is essential to define and understand different types of mental health disorders and how physiological signals in the body change in response to these disorders. With depression and anxiety being the most common mental disorders [1,2], these problems can affect individual welfare and economic productivity, affecting the ability of a person to participate in the labor force, which can contribute to societal costs because of production loss and reduced income taxation [1].

The costs associated with mental health are not only the losses incurred due to the inability of a person to contribute to the economy but also the costs of the resources used for the treatment [2], where direct costs can be attributed to outpatient visits and hospitalizations and indirect costs are losses to income [3]. Mental disorders diminish the supply of labor and capital, which results in poor output for the economy [3,4]. In 2019, a study by the “Institute of Health Metrics Association” indicated that 14.4% of global disabilities were because of mental health disorders and substance use disorders [1]. The World Economic Forum projected that by 2030, mental health illnesses will account for more than half of the global economic burden related to non-economic diseases at the cost of USD 6 trillion [2].

Mental disorders in the past were categorized broadly into psychoses or neuroses, where disorders with psychoses have severe symptoms such as delusions or hallucinations (bipolar or schizophrenia) and disorders with neuroses were less severe such as stress or trauma (anxiety or phobic) [5]. However, these days major health organizations in the world have clearly classified mental health disorders into a range of groups and subgroups. The *Diagnostic and Statistical Manual of Mental Disorders 5th edition* (DSM5), which was designed by the American Psychiatric Association to diagnose mental disorders [6], and the *International Classification of Diseases 11th Revision* (ICD-11), which was designed by the World Health Organization, has set the global reference for disorders by classifying it [7].

## 2. How Do Mental Health Disorders Affect the Human Body?

Mental health disorders do not only affect the functioning of the brain but can also affect the body and its functioning. It is important to understand how different types of disorders affect the physiological signals of the body since physiological signals can reflect the functioning of the human body and provide valuable information about the internal processes of the body. With respect to the human body, the signals can be classified into five categories such as electrical (ECG and EEG), mechanical (heart rate (HR), respiration rate), chemical (sweat and cortisol), and thermal (body temperature). To monitor mental health disorders from physiological signals, studies have used electrocardiogram, heart rate variability (HRV), electroencephalogram, galvanic skin response (GSR), and respiration rate [8,9].

An electrocardiogram measures the electric activity of the heart which is generated at the cellular level [10]. ECG signals can serve as a physiological biomarker for mental health disorders which aids in understanding the behavior of an individual [11]. ECG signals can be used to successfully detect psychiatric disorders like schizophrenia, bipolar disorders, and depression by detecting changes in cardiac activities through the signals [12]. Heart rate variability reflects the body’s ability to adapt to stress where higher HRV is linked to better emotional regulation and lower HRV is associated with mental disorders [13]. HRV also plays an important role in the diagnosis and treatment monitoring of neurodevelopmental disorders [14,15].

An electroencephalogram is used to measure the small electric potential generated by the neuronal actions in the brain, which is a non-invasive method for monitoring brain activity [16]. For disorders like schizophrenia, which is a type of neurological disability, EEG signals are used to detect altered neural activity, which aids in early detection and treatment [17,18]. Studies show that in EEG signals, band power and asymmetries in specific frequency ranges can be used as predictors for disorders [19]. Disorders like attention deficit hyperactivity disorder (ADHD), schizophrenia, and obsessive–compulsive disorder (OCD) show increased delta and theta powers while depression shows power across all bands. There are no significant differences with Post-traumatic Stress Disorder (PTSD), addiction, and autism [20].

Galvanic skin response measures the changes in skin conductance which can reflect the emotional state and stress level [21]. In a recent study, it was found that GSR can be used as a biomarker to understand adverse post-traumatic neuropsychiatric sequelae (APNS) among trauma survivors [22]. GSR can reflect the skin response differentiating between distress and eustress which could be used to detect distress levels that may lead to disorders [23].

Individuals with mental disorders (anxiety and depression) mostly suffer from symptoms like dyspnea and hyperventilation syndrome where they experience increased ventilation and altered breathing patterns [24]. Individuals with panic disorders exhibit larger variability in tidal volume and altered respiratory rates, which leads to chronic hypocapnia [25]. In a study conducted, it was found that individuals suffering from schizophrenia breathe more shallowly and rapidly while melancholics breathe more deeply and rapidly than normal individuals [26]. Cortisol is a steroid hormone that is produced by the adrenal glands and it plays an important role in the body’s response to stress [27]. Individuals who are associated with psychiatric disorders such as depression, bipolar disorders, and PTSD have high levels of cortisol levels [28,29]. The normalization of cortisol levels correlates with improvements in mental health; hence, it can be used as a biomarker [28]. Shahub S et al. [30] developed a non-invasive sensor that can detect cortisol in sweat; the sensor uses electrochemical impedance spectroscopy (EIS) to measure the concentration in the physiological range of 8–140 ng/mL. The sensor is designed with two gold electrodes on a nano-porous polyamide membrane substrate. The sensor’s response is dose-dependent and operates within a frequency of 100–500 Hz with inter-assay and intra-assay variations below 20%. The sensor could also be used in a smart wearable device to monitor the circadian rhythm.

## 3. Materials and Methods

### 3.1. Strategy for Searching

Smart textiles and mental health are emerging fields in smart wearables; hence, there were very few results when we searched for smart textiles and mental health. Hence, we had to look at the work carried out with the physiological signals of mental health disorders related to smart textiles. The keywords were “smart” and “intelligent” with a combination of “cloth”, “textile”, “fabric”, “apparel”, and “garment”, and with “e textiles” for the scope to be narrowed down to the smart textiles domain, and the physiological parameters were “Electrocardiogram”, “Electroencephalogram”, “Electrodermal activity”, “Skin conductance”, “Galvanic skin response”, “breathing rate”, “Cortisol”, “Respiratory rate”, and “Breathing pattern”. Searches were performed with the Web of Science and Scopus. Since the results were very few, the papers included had a range of 5 years from 2020 to 2024.

### 3.2. Criteria for Inclusion

The date of publication of the selected papers was between 2020 and 2024. Journal papers and conference papers were considered. The research direction for the paper was electrophysiological monitoring using smart textiles and the scope was limited to textile electrodes, smart clothes, and sensor technology with respect to textiles.

### 3.3. Criteria for Exclusion

**Figure 1:**
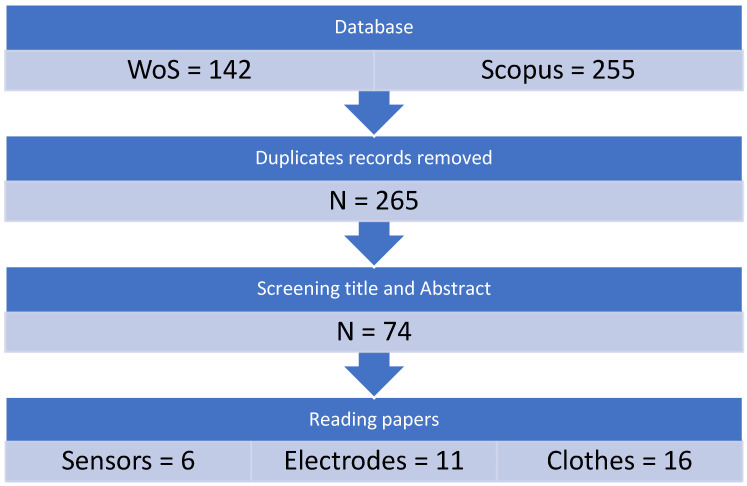
Flow diagram of the systematic search strategy.

Studies published in languages other than English were not included. The excluded studies included editorials, letters, highlights of research, press releases, review articles, technical papers, and reviews. Exempted were duplicate papers, related review papers, and publications unrelated to the subject. See Figure 1.

## 4. Textile Based Sensors

Sensors that can be integrated into fabrics are defined as textile-based sensors. These sensors are typically used to monitor physiological signals or detect exposure to the ambient environment. There are multiple ways by which these sensors can be integrated into the fabric, and some of the methods are textile fabrication, printing, and embedding. In textile fabrication, techniques such as knitting, weaving, or embroidery are employed [31,32,33]. For printing, methods such as screen printing, inkjet printing, or spray printing are used [34,35]. In the embedding process, the sensors are embedded into garments using a polymer matrix [36].

The materials used to create textile-based sensors could be made from conductive yarns, fiber optics, conductive polymers, piezoresistive textiles, and graphene-based inks [34,37,38,39,40]. Textile-based sensors offer several advantages when compared to conventional sensors, such as enhanced comfort, improved wearability, and seamless integration into garments [33,34,37,41]. They enable the unobtrusive monitoring of physiological and environmental parameters while maintaining flexibility and options to customize. Additionally, some of these sensors can be washable and durable, making them suitable for long-term use in various applications, including healthcare, sports, and human–machine interaction [36,40,42].

## 5. Critical Analysis of Previous Works

### 5.1. Developments in Sensor Capabilities

Sensor technology with respect to textiles involves integrating sensing capabilities directly into fabrics or textile-based structures to monitor various physiological parameters, environmental conditions, or mechanical deformations [32]. These technologies aim to create wearable devices that are comfortable, flexible, and unobtrusive, allowing for continuous and real-time monitoring [39,40].

When it comes to developing respiration rate sensors for smart textiles, Ali et al. [43] proposed using a stretchable fabric sensor impregnated with carbon black ink for conductivity and encapsulated by polydimethylsiloxane (PDMS) to enhance the stretchability of the sensor. This sensor works on the principle of piezoresistivity, where the electrical resistance of the sensor changes as it stretches and contracts with breathing. Di Tocco et al. [39] used conductive textiles with silver-coated yarn which was sewn into elastic bands placed around the chest to monitor breathing activity. The sensor measures changes in resistance due to the expansion and contraction of the rib cage during respiration; this sensor offers a comfortable way to monitor respiration rate, and they also used an Inertial Measurement Unit (IMU) to measure heart rate by detecting chest wall vibrations resulting from heartbeats using accelerometer and gyroscope data. These signals were processed using a Hilbert transform to extract the heartbeat envelope, which was then used for frequency analysis to determine the heart rate. Rumon et al. [32] explored knitted fabric structures using silver-based conductive yarn. These sensors work by measuring changes in resistance; as the knit structure stretches during breathing, different knit structures were tested for performance and the sensor was tested under various environmental conditions such as stretching and the presence of sweat to test the reliability. The main goals of these studies were that the materials used were safe for long-term skin contact and with a focus on long-term skin monitoring.

Traditional electrodes for EEG monitoring were performed with wet electrodes which require skin preparation and the possibility of the electrodes drying out with time causing signal degradation, skin irritations, and allergic reactions [40]. Mahmood et al. [44] employed a stretchable thin-film electrode that could be planted on the skin surface. They tested their EEG device on the back of the neck using a reference electrode on the mastoid process. They also demonstrated the use of two electrodes on the occipital lobe to capture alpha rhythms and steady-state visually evoked potential (SSVEP) signals.

They were able to monitor ECG using thin-film gold and graphene electrodes with an open mesh layout. These electrodes were placed on the chest and were designed to maintain intimate skin contact and minimize motion-related noise. They also tested their device for long-term monitoring, which was effective for 2 weeks. Naik et al. [45] developed a way by which cortisol levels can be monitored using electrochemical immunosensors with inkjet-printed graphene electrodes modified with gold nanoparticles and anti-cortisol antibodies. The sensor was able to detect the changes in current when cortisol binds to the antibodies, and it was able to operate within a range of 10 pM to 100 nM. See Table 1.

**Table 1:** Comparison summary of the sensors found.

Study	Objective	Signals	Textile Technology	Sensor Technology	Results
Ali et al. (2021) [43]	To develop a biocompatible and comfortable respiration rate sensor for e-textile and wearable applications.	RR	Stretchable fabric	Printed carbon black ink strain sensor based on resistance	Stable performance with variations from 160 Ω to 80 Ω, biocompatible and comfortable for daily use
Di Tocco et al. (2021) [39]	To develop a wearable system for the continuous and simultaneous monitoring of RR and HR, assessing feasibility in various scenarios.	RR and HR	Conductive textiles in elastic bands	Textile resistive strain sensors for RR, acceleration, and angular velocity for HR	Reliable RR and HR estimation in sitting, standing, and supine positions, and multi-sensor configuration shows promising results
Islam et al. (2022) [40]	To create a multifunctional e-textile platform for personalized healthcare, including biosensing, activity monitoring, and energy storage.	EEG	Screen printed	Graphene-based electrodes and textile supercapacitors	Comparable EEG performance to rigid electrodes, high wash stability, activity detection, and energy storage via supercapacitor with good stability
Mahmood et al. (2020) [44]	To develop a wireless, stretchable bioelectronics platform for portable, real-time physiological monitoring, including human–machine interfaces.	ECG, EEG, and EMG	Stretchable bioelectronics, hyper elastic elastomers, and printed and encapsulated electronics.	Nanomembrane sensor with multilayer electronic system	High-quality biopotential signals; real-time classification of ECG, EMG, and EEG data; wireless synchronization of multiple patches; and noise reduction
Naik et al. (2022) [45]	To develop an inexpensive, customizable microfluidic platform for single-use and continuous biomarker measurements in sweat.	Cortisol and glucose	Flexible microfluidic architecture	Inkjet-printed graphene and silver electrodes, gold nanoparticles (Au NPs) for cortisol, and Prussian blue (PB) for glucose detect changes in current	Cortisol detection limit of 10 pM, glucose detection limit of 10 μM, customizable for single and continuous measurements, and tested using synthetic skin
Rumon et al. (2023) [32]	To evaluate the performance of knitted stretch sensors for wearable health monitoring, covering structural design, material properties, and physiological signal detection.	RR and body movement	Knitted fabric structures (plain, stripe, and hybrid) with conductive yarn	Stretch sensors using silver-based conductive yarn, an electromechanical stretching system based on resistance	Detailed performance evaluation of knitted sensors demonstrated regulated respiration monitoring, effects of stretching on resistance were investigated, and durability and chemical stability were studied

### 5.2. Developments in Electrode Technologies

Electrode technology in the context of textile technology focuses on integrating conductive materials or structures into fabrics for biopotential monitoring [34,35,38]. The materials in the discussions are mostly conductive yarn which is incorporated into fabrics through weaving, knitting, or embroidery (silver-plated nylon and carbon-coated nylon) [31,38]. Conductive fabrics are made from metal wires woven with traditional textiles (woven conductive silver fabric and knitted jersey conductive fabric) [42]. Conductive polymers such as polystyrene sulfonate are applied via screen printing, offering good conductivity and biocompatibility [31,34]. Graphene electrodes are created by either dip coating, spray printing a layer of graphene on yarns, or using a laser on carbonized materials such as silk [35,46].

While conventional silver (Ag) or silver chloride (AgCl) electrodes provide good signal quality, they tend to dry out with time and they are not ideal for long-term monitoring as they cause skin irritation, rashes, and allergic dermatitis due to the adhesive gel, making them uncomfortable for daily and long-term monitoring [31,42,47], while textile electrodes, on the other hand, come with advantages such as comfortability, reduced skin irritation, breathability, flexibility, and integrability [31,38,42,46]. When it comes to signal quality for ECG monitoring, studies have shown that textile electrodes can achieve quality comparable to traditional Ag/AgCl electrodes with clear P, Q, R, S, and T complexes [35,37,46]. Studies have also shown that textile electrodes can maintain stable ECG recordings for extended periods of up to 24 h and they could be designed to minimize motion artifacts by providing stable ECG recordings even during physical activities like walking and running [46]. Studies have also found that some textile electrodes have low skin–electrode contact impedance which is similar to traditional gel-based electrodes [35,46]. Ozturk et al. [35] developed a textile electrode that is placed on the arm, providing a convenient alternative to chest-based electrode placement. In this arrangement, it is especially useful for long-term monitoring because it allows for freedom of movement and may be less affected by motion artifacts.

Traditionally, EDA monitoring devices made use of rigid electrodes such as rings, wristbands, or wearable sensors which are often used to measure the tonic skin conductance (autonomic arousal) and may not have enough sampling rate to capture the phasic data (short-term arousal) [48]. On the other hand, textile electrodes can be effectively used to capture both tonic and phasic responses [34]. The study by Janusz et al. [49] proposed two different types of electrodes which were created from polyethylene-co-vinyl acetate (PEVA) and polydimethylsiloxane elastomer (PDMS), respectively. Their goal was to measure respiration using two principles fiber strain method and bioimpedance method where the PDMS showed more promise in detecting breathing rate with better accuracy, signal to noise ratio (SNR), and versatility. Tseghai et al. [50] developed a novel electrode for EEG monitoring that could replace the traditional gold cups or Ag/AgCl comb electrodes, thus avoiding the need for pre-treatment, for example, the application of conductive gels and head shaving. The electrodes were created using electrically conductive hook fabric which could establish direct contact with the skin and can penetrate through the hair. The electrode was found to have lower skin-to-electrode impedance, higher signal to noise ratio, and slightly better performance than standard electrodes. See Table 2.

**Table 2:** Comparison summary of the electrodes found.

Study	Objective	Signals	Textile Technology	Sensor Technology	Results
Abd-Elbaki et al. (2023) [46]	To develop robust, self-adhesive, and anti-bacterial electrodes for electrophysiological monitoring.	ECG	Silk-based Laser-Induced Graphene (LIG) using carbonization method	Self-adhesive, anti-bacterial LIG silk fabric electrodes	Excellent characteristics compared to other electrodes, stable ECG recordings for 24 h, low sheet resistance, low contact impedance, mechanical robustness, and anti-bacterial properties
Alizadeh Meghrazi et al. (2020) [38]	To develop a wearable, non-invasive system for continuous HRV monitoring from the waist; integrated into garments.	ECG and HRV	Knitted textile sensors (silver- and carbon-based)	Multichannel ECG band with four sensors on the waist	Reliable ECG signals from the waist; comparable QRS complex to chest recordings; improved R-peak detection with probabilistic approach; and tested with sitting, standing, and jogging
Arquilla et al. (2021) [37]	To design, develop, and evaluate woven textile electrodes to detect the full ECG waveform (P, Q, R, S, and T peaks).	ECG	Woven textile electrodes	Electrodes of different sizes, patterns, and thread types	Woven electrodes are viable for ECG, silver thread may be less prone to false positives, and electrode design affects signal quality
Janka et al. (2022) [48]	To develop dry textile electrodes for skin conductance measurement; integrated as an invisible part of clothing.	EDA	Embroidered electrodes	Copper- and silver-based fabrics dry electrodes	Copper and densely embroidered silver thread electrodes showed the best response. Electrodes with dense surfaces have low contact resistance.
Janusz et al. (2022) [49]	To develop an RR sensor that can be embedded into a wearable garment.	RR	Flexible, multi-material fibers (PEVA and PDMS)	Conductive fibers using fiber strain and bioimpedance methods	PDMS fiber outperformed PEVA fiber in detecting breathing rate. Bioimpedance shows promise for accuracy and comfort.
Ozturk et al. (2023) [35]	To develop wearable, single-arm ECG systems with graphene textile electrodes for health monitoring.	ECG and HRV	Graphene-coated textiles (dip-coated and spray-printed)	Single-arm ECG prototype with graphene electrodes	High correlation with standard electrodes (up to 98%), spray-printed better than dip-coated, accurate heart rate detection, R-peak detection, and minimal RMS noise increase
Rajanna et al. (2020) [42]	To evaluate conductive fabrics as electrodes for ECG acquisition, with reduced skin-contact impedance.	ECG	Woven conductive silver and knitted jersey fabrics	Dry textile electrodes with polymer foam	Reduced skin-contact impedance (less than 1 MΩ/cm^2^), silver electrodes showed better fidelity, and knitted jersey showed good resistance motion artifacts
Ren et al. (2021) [47]	To study e-textile-based dry electrodes for contact ECG recording.	ECG	Copper and e-textile dry electrodes	Electrodes with different shapes and areas	Electrodes with larger sizes provide higher average-to-variation ratio values
Sinha et al. (2020) [34]	To develop integrated sensors on a t-shirt for the continuous and simultaneous measurement of ECG, EMG, and EDA.	ECG, EMG, and EDA	Polymer screen printing	PEDOT: PSS electrodes	Electrodes recorded simultaneous ECG, EMG, and EDA. Higher signal amplitude than Ag/AgCl electrodes (EMG), stable after washing.
Tseghai et al. (2023) [50]	To develop a dry EEG electrode from conductive hook fabric to overcome the limitations of the existing electrodes.	EEG	Conductive hook fabric	Dry EEG electrode from conductive Velcro tape and textile-based knitted-net EEG bridge	Lower skin-to-electrode impedance when compared to Ag/AgCl electrodes, higher signal to noise ratio compared to cup electrodes, and doubling the size of the hook results in a 17.3% increase in SNR
Zhang et al. (2022) [31]	To design and fabricate woven fabric electrodes for ECG monitoring; optimize parameters for integration into smart textiles.	ECG	Woven fabric electrodes (silver-plated nylon)	Electrodes with different diameters, weaves, and weft densities	Fabric electrodes showed better ECG waveforms than standard medical electrodes in dynamic states. Improvement by 86.7%.

### 5.3. Developments in Smart Clothes Fabrication

Smart clothing refers to garments that integrate technology to monitor various physiological signals. These garments incorporate sensors, electrodes, and conductive materials directly into the fabric to collect data related to the wearer’s health and well-being [51,52,53]. These garments can also be more comfortable and less obtrusive than traditional monitoring devices, which allows for continuous wear and data acquisition [41,51,54]. Hence, these garments can enable the long-term, continuous monitoring of physiological parameters such as electrocardiogram, respiration rate, and electrodermal activity, also providing instant and detailed results on vital changes that can be communicated wirelessly to a system for analysis [41,51,55,56].

Smart garments use textile-based electrodes mostly made from conductive materials such as silver yarns, conductive elastomeric fibers, or conductive fabric [33,41,51,52]. To detect the heart’s electrical activity, these electrodes are placed strategically on the body to capture ECG signals. For single-lead systems, some smart garments focus on a single lead for basic heart rhythm analysis where these configurations are often sufficient for detecting heart rate and rhythm abnormalities [51,52]. In multi-lead systems, there is more than one lead configuration that can support up to 12 leads for ECG monitoring, which provides a more detailed picture of the heart’s electrical activity, and it can be comparable to a standard clinical ECG [52,57,58] where these configurations are useful for detecting a wider range of cardiac conditions and localizing arrhythmias [52]. Chen et al. [54] developed a noncontact system to measure ECG signals by using capacitive electrodes that do not directly touch the skin. These systems measure the signals through a fabric layer and are suitable for long-term monitoring with reduced skin irritation.

Respiration monitoring with smart clothes can be measured in different ways with piezoresistive sensors which are placed on the chest or abdomen to measure the expansion and contraction of the body during breathing, which correlates with the breathing cycle [36,55,59,60]. Capacitive sensors, on the other hand, use changes in capacitance to detect breathing patterns, which consist of two electrodes separated by a dielectric material with the human body acting as a part of the capacitive structure. As the body expands and contracts during breathing the capacitance changes, which can be measured and is used to determine the respiratory rate [51]. Garments with textile-based antenna sensors use a meander dipole antenna made of silver-coated nylon thread integrated into a garment. The respiratory signal is extracted from the received signal strength indicator (RSSI) emitted from the antenna-based sensor. The RSSI values change during breathing due to chest and abdominal deformation [61]. These sensors are made with conductive threads stitched into clothing. As the chest expands and contracts during breathing, these sensors are stretched, which results in a change in resistance that can be measured [53,60].

Ferreira et al. [56] developed a smart sock form factor to address challenges in signal saturation and scalability for foot-based EDA monitoring which resulted in a high correlation (up to 95%) between foot and hand EDA signals (gold-standard measurement). López-Larraz et al. [62] developed a proof-of-concept garment capable of measuring EEG signals completely out of thread, fabrics, and smart textiles; the EEG electrodes were placed on the scalp according to the International 10/20 system which uses specific locations such as F3, F4, P3, and P4. The results can be compared to the dry electrodes but suffer from artifacts due to poor contact impedance. Zeng et al. [63] developed a textile cap with EEG electrodes which was designed in a 4-channel configuration to detect emotions. The smart garment was able to classify emotions with an accuracy of 72%, proving that this system could be used as an alternative solution in the market, enabling long-term monitoring. See Table 3.

**Table 3:** Comparison summary of the smart clothes found.

Study	Objective	Signals	Textile Technology	Sensor Technology	Results
Al Rumon et al. (2023) [55]	To create a smart T-shirt for guided breathing exercises	RR and HR	E-textile sensors integrated into a T-shirt	Three-channel e-textile respiration sensors with an analog front-end board	Average respiration event detection accuracy was around 98%. HR was validated against a 3-lead standard ECG, achieving 99% accuracy. RR-HR correlation analysis showed an R-square value of 0.987.
Chen et al. (2024) [54]	To design a clothing system with noncontact electrodes for ECG monitoring	ECG	Woven cotton, knit fabric, and high elastic lining fabrics	Noncontact electrodes using copper plates	ECG signal quality was affected by the fabric type, thickness, and moisture. The cotton fabric had the least impact on amplitude attenuation. Signal correlation greater than 95% like Ag/AgCl electrodes.
El Gharbi et al. (2023) [61]	To monitor breathing patterns using an embroidered antenna-based sensor	RR	Embroidered antenna-based sensor in a T-shirt using silver-coated nylon thread	Textile antenna-based sensor	Various types of breathing patterns such as Eupnea, Biot, and Cheyne–Stokes were recognized by the respiratory signals acquired wirelessly via Bluetooth with RSSI.
Eskandarian et al. (2022) [41]	To develop flexible, breathable, and washable dry textile electrodes for long-term monitoring	ECG, EOG	3D knit conductive elastomeric filaments (CEFs)	Dry textile electrodes made of CEFs	Comparable fidelity to gel electrodes. Electrodes were resistant to 30 wash and dry cycles.
Ferreira et al. (2024) [56]	To create a smart sock for EDA monitoring	EDA	Conductive Lycra electrodes in a smart sock	Optimized EDA sensor with a wider measurement range	The improved prototype reduced signal saturation and had a latency between foot and hand EDA signals of 1.53 ± 0.86 s.
Fink et al. (2021) [57]	To develop ECG garments with textile-based dry electrodes	ECG	Knitted fabrics, polyamide with elastane, and mesh fabric	Textile-based dry electrodes	Dry electrodes can suffer from high or unstable skin–electrode impedance. The garment design included an adjustable chest panel.
Fouassier et al. (2020) [52]	To evaluate a smart T-shirt for ECG acquisition	ECG	Smart T-shirt with textile electrodes made of silver yarns and hydrogel pads	Textile electrodes with hydrogel pads	ECG signal quality was comparable to Holter recordings with appreciation of cardiac rhythm in 100% of the recordings during rest. No skin irritation was observed.
Lee et al. (2022) [51]	To develop a smart clothing system for long-term monitoring of ECG and respiration	ECG, RR	Conductive fiber traces and embedded sensors	Fabric-based dry electrodes for ECG and a capacitive transducer for respiration	High-quality ECG signals with a QRS complex detection sensitivity of 99.86%. Respiration transducer accuracy of 98.74%.
Linti et al. (2022) [58]	To develop a comfortable smart garment for ECG monitoring	ECG	Embroidery	Textile electrodes	QRS complexes were correctly identified. Electrodes showed comparable results to conventional adhesive electrodes.
López-Larraz et al. (2023) [62]	To develop a textile-based EEG sensor	EEG	Embroidery	3-strand silver-coated nylon conductive yarns	Under favorable conditions, the garment provided comparable measurements to a metal-based EEG system.
Martini et al. (2022) [53]	To create a respiratory rate monitoring garment using stitched sensors	RR	Thread stitched	Conductive thread sensors operate based on changes in resistivity	A linear relationship between resistance and displacement in stitched sensors samples.
Massaroni et al. (2020) [59]	To assess sensors for the performance of respiratory monitoring during exercise	RR	Elastic bands, knitted fabric with silver, and spandex	Piezoresistive sensors connected to Wheatstone bridges and instrumentation amplifiers	Capable of estimating average and breath-by-breath respiratory rate during walking and running.
Mouckova et al. (2020) [60]	To create a textile-integrated respiratory rate monitoring system	RR	Electrically conductive silver yarns knitted into a non-conductive textile structure	Textile sensor based on changes in electrical resistance due to the stretching of conductive yarns	The system was compared with a reference spirometer and tested for repeatability. The system can be used to monitor respiratory rate.
Romano et al. (2024) [36]	To formulate the feasibility of a t-shirt for respiratory monitoring	RR	Sensors embedded in a t-shirt using a polymer matrix	Piezoresistive textile sensors	Sensors were metrologically characterized with sensitivities of 9.4 and 9.1, and a mean absolute error of 0.32 bpm was achieved.
Schauss et al. (2022) [33]	To verify a wearable ECG monitoring system for women	ECG	Woven textile electrodes integrated into a garment	Textile electrodes (steel yarn)	R peaks were perfectly detected, with an overall signal quality equivalent to adhesive electrodes. A comfortable and long-term solution to cardiac monitoring.
Zeng et al. (2020) [63]	To develop a comfortable and user-friendly textile-integrated EEG cap	EEG	Elastic fabric and ultra-soft urethane gel holder	4-channel dry comb electrodes EEG cap integrated with textiles	A strong correlation (average of 0.857) with signals from commercial wet electrodes. The device was comfortable, lightweight, soft, and aesthetically pleasing.

## 6. Discussion

The review, conducted in a thorough manner, demonstrates the current state of the art in smart textile technologies for mental health monitoring, emphasizing substantial advancements in sensors, electrodes, and smart garments. The taxonomy presented organizes these technologies into three primary categories: sensor capabilities, advances in electrode technology, and smart garment manufacturing. This framework serves to facilitate comprehension of the various technological solutions and assist in identifying pivotal areas for future enhancement. The ensuing discussion addresses prevailing challenges, interdisciplinary perspectives, and future applications in the context of these technologies.

### 6.1. Challenges and Possibilities in Smart Textiles for Mental Health

#### 6.1.1. Applications in Mental Health

Smart textiles have shown immense potential in addressing various aspects of mental health, spanning prevention, monitoring, and intervention. Specific applications include the following:

- Prevention: Wearable smart textiles equipped with sensors for the continuous monitoring of stress indicators (e.g., heart rate variability and cortisol levels) can aid in preventing chronic stress and burnout by providing early warnings and actionable insights [13,27].
- Occupational Stress: In high-stress environments, such as healthcare or emergency services, smart garments can continuously monitor physiological stress markers. Studies, such as those utilizing HRV data, have demonstrated their utility in identifying patterns of occupational stress and promoting timely interventions [13,14].
- Developmental Disorders in Children: Textile-based solutions like EEG caps can facilitate the early detection of conditions like ADHD by monitoring neural activity during daily activities. This non-invasive, comfortable approach supports early intervention strategies [16,19].
- Elderly with Depression and Loneliness: Smart textiles can assist in monitoring the physiological signs of depression in elderly populations. Combined with Artificial Intelligence (AI)-based emotion detection, these tools can provide critical insights for caregivers and improve mental health outcomes [27,28].
- Social Isolation: Smart garments can integrate vibrational or haptic feedback to encourage interaction or relaxation, addressing loneliness in various demographic groups [29].

The reviewed literature indicates that while these applications are promising, challenges such as accuracy under real-world conditions, sensor robustness, and user comfort must be addressed to achieve widespread adoption.

#### 6.1.2. Integration with Artificial Intelligence

AI integration enhances the capabilities of smart textiles by providing real-time data analysis, predictive insights, and personalized interventions. The current advancements in AI-driven mental health applications include the following:

- Emotion Detection: AI algorithms trained on physiological data collected by smart textiles can classify emotional states with reasonable accuracy (e.g., 72% accuracy in EEG-based emotion detection) [19,63].
- Personalized Feedback: By combining wearable data with machine learning, users can receive tailored recommendations, such as relaxation exercises or breathing techniques [13,63].
- Anomaly Detection: AI models can identify deviations from baseline physiological states, flagging potential mental health concerns [14,20].

Despite these advancements, ethical concerns regarding data ownership, privacy, and potential biases in AI models require careful consideration, as discussed in the following section.

#### 6.1.3. Ethical and Privacy Concerns

The integration of smart textiles with mental health monitoring raises critical ethical and privacy issues:

- Data Privacy: Continuous data collection necessitates robust encryption and secure storage solutions to protect sensitive user information. Privacy-preserving methods like differential privacy can mitigate risks [30].
- Informed Consent: Users must be fully aware of how their data will be used, shared, and stored. Transparent data usage policies are essential [30].
- Bias and Fairness: AI models used in smart textiles should be trained on diverse datasets to avoid bias, ensuring equitable outcomes across different populations [14].

The literature emphasizes the importance of adopting a “privacy by design” approach and compliance with regulations such as the General Data Protection Regulation (GDPR) to address these challenges effectively.

#### 6.1.4. Usability and Adoption Barriers

The success of smart textiles depends on their usability and user acceptance. The key barriers identified include the following:

- Comfort and Design: Ensuring long-term wearability without causing discomfort or skin irritation is critical. Studies have shown promising results with conductive fabric electrodes and stretchable bioelectronics [46,47].
- Cost: High manufacturing costs limit accessibility, especially in low-resource settings. Scalability and cost-efficient production methods, such as the inkjet printing of sensors, can address this [38,45].
- Material Safety and Skin Compatibility: Smart textiles are worn for extended periods, making material safety a crucial consideration.
- Biocompatibility and Dermatological Safety: Materials used in sensorized garments should be hypoallergenic and free from irritants to prevent skin reactions. Common concerns include contact dermatitis and hypersensitivity reactions to conductive materials such as silver-coated fibers or metallic nanoparticles [46,47].
- Potential Allergic Reactions: Studies indicate that prolonged exposure to certain conductive polymers, adhesives, or textile coatings can cause skin irritation, particularly in individuals with pre-existing skin conditions such as eczema [31,32].
- Alternative Materials: The use of organic and natural fibers, antimicrobial coatings, and breathable fabrics can enhance skin tolerance while maintaining sensor effectiveness [46,47].
- Ease of Use: User-friendly interfaces and seamless integration with the existing devices are essential for widespread adoption. Technologies like Bluetooth-enabled smart garments demonstrate potential in this area [51].

#### 6.1.5. Sustainability and Environmental Impact

As smart textiles gain popularity, their environmental footprint becomes a concern. Potential avenues include the following:

- Biodegradable Materials: The development of eco-friendly, biodegradable sensors and fabrics to reduce waste [46].
- Energy Efficiency: Designing low-power sensors and incorporating renewable energy sources, such as solar-powered wearables [51].
- Recyclability: Modular designs that allow the easy separation of electronic components for recycling can minimize environmental impact [33].

Future research should prioritize sustainability to ensure the long-term viability of smart textiles in mental health applications.

#### 6.1.6. Personalization of Smart Textile Solutions

Personalized approaches are critical for addressing the diverse needs of mental health populations. Smart textiles can carry out the following:

- Adapt to Individual Baselines: AI models can learn and adapt to individual physiological patterns, improving the accuracy of anomaly detection [63].
- Support Diverse Use Cases: From guided breathing exercises to real-time emotion monitoring, personalization enhances the relevance and effectiveness of interventions [55].
- Tailor Fit and Functionality: Customizable garments that cater to specific user preferences (e.g., fabric type and fit) improve adherence [51,56].

Personalization requires collaboration between engineers, healthcare professionals, and end-users to align technological capabilities with practical needs.

#### 6.1.7. Regulatory and Standardization Challenges

The lack of standardized guidelines poses significant challenges to the widespread adoption of smart textiles:

- Regulatory Approval: Smart textiles intended for medical applications must comply with rigorous regulatory frameworks, such as Food and Drug Administration (FDA) or Conformité Européenne (CE) certifications [38].
- Interoperability Standards: Establishing standards for data formats and communication protocols is essential to ensure compatibility across devices and platforms [33].
- Ensuring adherence to medical-grade textile standards, such as the International Organization for Standardization (ISO) 10993 for biocompatibility or FDA/CE certification for wearable medical devices, is essential for minimizing health risks [41,42].
- Quality Assurance: Standardized testing protocols for sensor accuracy, durability, and safety are critical to gaining user and regulatory trust [46].

Efforts to create industry-wide standards and streamline approval processes will play a pivotal role in advancing smart textile technologies.

#### 6.1.8. Smart Textiles in Digital Therapeutics (DTx)

The rise in digital therapeutics (DTx) has transformed mental healthcare by enabling evidence-based interventions delivered via digital platforms. Smart textiles can enhance DTx by the following ways:

- Facilitating Cognitive Behavioral Therapies (CBTs): Wearables can provide real-time feedback during CBT exercises, such as stress reduction techniques or mindfulness practices. For instance, smart garments can detect physiological changes during guided breathing sessions and adjust exercises accordingly [55,59].
- Remote Monitoring: Smart textiles enable continuous monitoring, ensuring adherence to DTx protocols and providing clinicians with actionable insights [56].
- Improving Engagement: Interactive features, such as haptic feedback, can make DTx sessions more engaging and effective [61].

The integration of smart textiles with DTx represents a significant step towards holistic, technology-driven mental healthcare, as evidenced by the growing body of research in this field.

### 6.2. Challenges in Manufacturing Smart Textiles

Despite more than 25 years of existence of intelligent medical devices, as they were called in 1997, sensorized garments have seen minimal commercial success. One of the key challenges lies in the textile manufacturing of smart wearable sensing garments. Despite decades of research and development, comprehensive information on textile manufacturing methods for smart textiles remains scarce, as highlighted by a recent literature review by Ramos, A. et al. [64]. However, recent work by Majumder et al. [65] provides valuable insights into the potential manufacturing techniques for smart textiles, which often combine traditional and advanced methods. 

#### 6.2.1. Traditional Manufacturing Techniques

- Knitting and Weaving: Commonly used to incorporate conductive threads or yarns into fabric structures, enabling flexibility and durability for applications like resistance temperature detectors (RTDs) and biopotential sensors (e.g., ECG and EEG).
- Embroidery: Conductive threads are embroidered onto fabrics to create circuits or sensor patterns, such as pressure-sensitive elements or respiration rate monitors while maintaining aesthetic and structural integrity.
- Dip Coating: Layer-by-layer (LbL) assembly deposits functional materials, such as conductive polymers or nanocomposites, onto textiles, creating durable and washable smart fabrics.

#### 6.2.2. Advanced Manufacturing Techniques

- Vapor-Phase Polymerization (VPP): Coating textiles with conductive polymers like PEDOT improves electrical properties and enables applications such as tactile sensors.
- Spray Coating: The precise application of materials, minimizing waste, to create components like supercapacitors and humidity sensors.
- Screen Printing: Applying conductive inks and pastes onto textiles, widely used for fabricating wearable ECG electrodes and flexible supercapacitors.
- Electrospinning: Producing ultrafine fibers that incorporate nanomaterials like carbon nanotubes (CNTs) or graphene for use in piezoelectric nanogenerators and drug delivery systems.
- Carbonization: Transforming fibers into highly conductive and flexible materials suitable for wearable electronic skins and pressure sensors.
- Hydrogels: Integrating conductive hydrogels with textiles to create fibers that are stretchable, self-healing, and responsive to environmental stimuli.

#### 6.2.3. Manufacturing Challenges

Combining traditional and advanced methods is often a custom, function-specific process tailored to the requirements of the sensorized garment. While this flexibility enables innovation, it also presents significant barriers to scaling production beyond research settings into commercialization. Issues such as scalability, durability, and cost-effectiveness hinder the widespread adoption of smart textiles, limiting their potential in applications like personalized health monitoring.

To address scalability concerns, several key areas require further attention:

- Process Standardization: The current smart textile manufacturing lacks uniformity in production techniques, leading to inconsistencies in product quality. Standardizing fabrication methods can enhance efficiency and reproducibility.
- Integration of Electronic Components: The seamless embedding of electronic components into textiles remains complex. Scalable methods such as roll-to-roll printing and automated stitching processes need further development.
- Durability and Washability: Ensuring that smart textiles maintain their functionality after repeated washing cycles is crucial for commercialization. Research on protective coatings and robust conductive fibers is ongoing.
- Cost-Effective Production: Reducing the cost of materials and optimizing production lines are essential for market competitiveness. Developing scalable low-cost alternatives such as inkjet-printed sensors and biodegradable electronics could facilitate wider adoption.

## 7. Conclusions

The most salient finding of this work is the demonstration of the potential of smart textiles to revolutionize mental health monitoring. The ability to collect physiological data continuously and non-invasively positions these technologies as key tools in the transformation of mental healthcare towards a more proactive and personalized model. Additionally, the comfort offered by these wearables allows their integration into daily life, a feat not possible with traditional monitoring technologies.

However, it is crucial to acknowledge the necessity of overcoming technical challenges, particularly those on the elimination of artifacts and the enhancement of material durability, if these solutions are to achieve viability in the market. Moreover, the development of such products that are not only functional but also safe and accessible necessitates interdisciplinary collaboration between engineers, psychologists, and textile designers. The future of smart textiles in mental health monitoring and management holds immense potential, signifying a substantial advancement in the realm of digital health.

Future studies should focus on advancing materials with improved biocompatibility and durability, optimizing AI-driven analytics for more accurate mental health assessments, and developing scalable, cost-effective manufacturing processes. Additionally, investigating sustainable and biodegradable sensor materials could help minimize the environmental footprint of smart textiles. Addressing these research directions will accelerate the adoption of smart textiles in mental health applications and enhance their effectiveness in real-world scenarios.

## Data Availability

No new data were created or analyzed in this study. Data sharing is not applicable to this article.

## Abbreviations

The following abbreviations are used in this manuscript:

RR	respiration rate
EGG	electroencephalogram
EDA	electrodermal activity
ECG	electrocardiogram
WoS	Web of Science
WHO	World Health Organization
USD	United States Dollar
DSM5	*Diagnostic And Statistical Manual of Mental Disorders 5th Edition*
ICD-11	*International Classification of Diseases 11th Revision*
HR	heart rate
HRV	heart rate variability
GSR	galvanic skin response
ADHD	attention deficit hyperactivity disorder
OCD	obsessive–compulsive disorder
PTSD	Post-traumatic Stress Disorder
APNS	adverse post-traumatic neuropsychiatric sequelae
EIS	electrochemical impedance spectroscopy
PDMS	Polydimethylsiloxane
IMU	Inertial Measurement Unit
SSVEP	steady-state visually evoked potential
Au NPs	gold nanoparticles
PB	Prussian blue
Ag	silver
AgCl	silver chloride
PEVA	Polyethylene-Co-Vinyl Acetate
PDMS	Polydimethylsiloxane Elastomer
SNR	signal to noise ratio
LIG	Laser-Induced Graphene
PEDOT	Poly (3,4-Ethylenedioxythiophene)
PSS	Poly (Styrene Sulfonate)
RSSI	received signal strength indicator
EOG	Electrooculogram
CEFs	conductive elastomeric filaments
AI	Artificial Intelligence
GDPR	General Data Protection Regulation
FDA	Food And Drug Administration
CE	Conformité Européenne
ISO	International Organization for Standardization
DTx	digital therapeutics
CBTs	Cognitive Behavioral Therapies
RTDs	resistance temperature detectors
LbL	Layer-By-Layer
VPP	Vapor-Phase Polymerization
CNTs	carbon nanotubes
